# Intelligent Manufacturing and Carbon Emissions Reduction: Evidence from the Use of Industrial Robots in China

**DOI:** 10.3390/ijerph192315538

**Published:** 2022-11-23

**Authors:** Hao Lv, Beibei Shi, Nan Li, Rong Kang

**Affiliations:** 1 School of Economics & Management, Northwest University, Xi’an 710127, China; 2 National and Local Joint Engineering Research Center of Carbon Capture and Storage Technology, Xi’an 710069, China; 3 Shaanxi Key Laboratory for Carbon Neutral Technology, Xi’an 710069, China; 4 Carbon Neutrality College (Yulin), Northwest University, Xi’an 710127, China

**Keywords:** intelligent manufacturing, climate governance, energy consumption, carbon emissions

## Abstract

Driven by the information technology revolution, using artificial intelligence to promote intelligent manufacturing while achieving carbon emissions reduction is increasingly the focus of international attention. Given this, based on the fact that China’s industrial manufacturing is more intelligent, this paper uses industrial sector data and robot data from 2000 to 2017 to examine the impact of intelligent manufacturing on industrial carbon dioxide emissions and to discuss its internal mechanism. The research found that intelligent manufacturing significantly inhibits carbon dioxide emissions in the industrial sectors. The emission reduction effect is more obvious in industries with higher carbon emissions and intelligence. The mechanism test shows that intelligent manufacturing mainly achieves industrial emission reduction by reducing fossil energy consumption in the production process and improving energy use efficiency. The research findings of this paper provide favorable evidence for using new technologies, such as artificial intelligence, to achieve carbon emissions reduction, and validate the importance of intelligent manufacturing in tackling climate change in the future. It provides an essential reference for developing countries to use artificial intelligence for their carbon emissions reduction goals.

## 1. Introduction

Climate change has led to more frequent extreme weather events, which have threatened economic activities such as agriculture, forestry, animal husbandry, and fishing [1], aggravated the spread of diseases [2], and threatened economic growth [3] and public health [4]. Many studies on climate change show that carbon dioxide emissions from energy consumption are the leading cause of climate change [5]. Therefore, reducing carbon dioxide emissions has become a common concern of all countries to lessen the impact of climate-related disasters. However, considering the differences in the stages of economic development faced by developed and developing countries, different countries should play different roles in reducing carbon emissions [6]. Although there are many disputes between developed and developing countries about the contributions of carbon emission reduction [7], it is important to note that developing countries are currently playing an essential role in achieving carbon emission reduction and tackling climate change [8].

**Figure 1:**
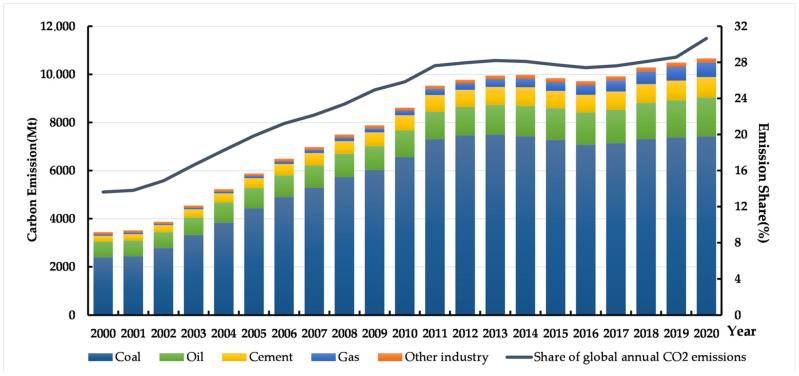
China’s CO_2_ emissions by flue type and share of global CO_2_ emissions. Source: Global Carbon Project.

As the world’s biggest carbon emitter, China accounts for about 30% of the world’s carbon dioxide emissions, 90% of which are caused by fossil energy consumption [9]. Figure 1 shows China’s CO_2_ emissions by flue type and the share of global CO_2_ emissions. Since the reform and opening-up, China’s industrial added value has increased approximately 60-fold from USD 96.05 billion in 1978 to USD 577 billion in 2020 (Constant 2015 USD) [10]. The rapid outward development of China’s economy mainly relies on high investment and high energy consumption, resulting in a large amount of crude input of fossil energy, leading to a large amount of carbon dioxide emissions [11]. At the same time, rapid economic growth has led to dramatic declines in environmental quality [12]. To actively and effectively address the issue of climate change, at the general debate of the United Nations General Assembly in September 2020, President Xi Jinping declared that China would scale up its Nationally Determined Contributions (NDCs) by adopting more vigorous policies and measures and aims to have CO_2_ emissions peak before 2030 and achieve carbon neutrality before 2060. The timespan from carbon peak to carbon neutrality that the Chinese government promised is much shorter than what many developed countries might take [13]. Therefore, it is very challenging for the Chinese government to achieve these goals on time.

Much to our gratification, the rise of artificial intelligence injects new impetus into China’s economic growth and provides new solutions for improving China’s ecological environment [14]. According to the statistics, China accounted for nearly one-fifth of global private investment funding in 2021, attracting USD 17 billion for AI (Artificial Intelligence) start-ups [15]. Stanford University’s AI Index, which assesses AI advancements worldwide across various metrics in research, development, and economy, ranks China among the top three countries for global AI vibrancy. Facts have proved that China has built a solid foundation to support its AI economy [16]. The development of artificial intelligence has become a new trend in China’s current economic growth, and new industries represented by new information technologies such as big data, cloud computing, and mobile internet have formed. Then, while the information technology revolution promotes the vigorous development of intelligent manufacturing, can it bring about the dual effects of economy and ecology? Can it significantly improve the production efficiency of various industries and promote economic growth? Can it reduce energy consumption and achieve carbon emission reduction? Can it be an effective means to achieve carbon neutrality? It is worth studying whether intelligent manufacturing can reduce carbon emissions in industrial sectors and by what means to achieve carbon reduction in all industries. To this end, based on the industry data and robot production data from 2000 to 2017, this paper examines the impact of intelligent manufacturing on industrial carbon dioxide emissions and discusses its internal mechanism.

## 2. Literature Review

Progress in information technology has promoted extensive changes in the economic growth pattern and development structure. It also has significantly impacted how energy is produced and consumed in countries around the world. However, the effects of information technology on energy consumption are complex. On the one hand, information technology can reduce energy consumption in products and processes through its direct application, potentially offering considerable improvements in energy efficiency for the transport, buildings, and industry sectors. On the other hand, information technology will tend to increase electricity’s importance in the economy. The prevalence of more devices could cause significant net increases in energy use. At the same time, AI technologies based on big data and cloud computing are widely used in industry and profoundly impact electricity and energy use. Scholars have conducted many studies to assess the potential impact of information technology and AI on energy use.

### 2.1. Information Technology and Energy Consumption

Many scholars have studied fossil energy consumption in industrial production and found that intelligent manufacturing enabled by information technology can decrease the demand for fossil energy consumption and improve energy use efficiency. Schulte et al. (2016) used a cross-country cross-industry panel data set covering 13 years, 10 OECD countries, and 27 industries and found that information technology reduced total and non-electric energy demand, but electrical energy demand was not significantly affected [17]. Usman et al. (2020) analyzed the impact of ICT (Information and Communication Technology) on economic performance and energy consumption in selected South Asian economies from 1990 to 2018. The study shows that in both the short and long term, ICT helped reduce India’s energy consumption and significantly improved the energy efficiency of Indian industries [18]. Ishida et al. (2015) used the Autoregressive Distributed Lag (ARDL) boundary test approach to assessing the long-term relationship between ICT, energy consumption, and economic growth in Japan. The study shows that ICT investment can equally contribute to a modest reduction in Japan’s energy consumption but not to an increase in GDP (Gross Domestic Product) [19]. Bastida et al. (2019) assessed the potential of ICT-based interventions in households to decrease electricity usage, and improve energy efficiency, finding that ICT-based effects on consumer behavior can reduce household final electricity consumption by 0–5% [20].

A few scholars have found that progress in information technology has not always contributed to lower energy consumption. Saidi et al. (2017) investigated the impact of information communication technology (ICT) and economic growth on electricity consumption for a global panel of 67 countries using a dynamic panel data model. They found that ICT and economic growth increased electricity consumption [21]. Zhou et al. (2018) used a three-tier structural decomposition analysis (SDA) approach to analyze the main drivers behind China’s change in energy intensity. They found that while higher energy efficiency in the ICT sectors led to a slight decrease in energy intensity, structural changes in ICT investments increased energy intensity. The spread of ICT products increased energy consumption in the production process [22]. Lange et al. (2020) used an economic-environmental analytical model to discuss ICT’s direct and indirect effects on energy consumption and energy efficiency. The study showed that ICT brought additional energy consumption instead of saving energy. The energy-increasing impact of information technology (direct effect and economic growth) was more significant than the energy-reducing effect (energy efficiency improvement and sectoral changes) [23].

### 2.2. Information Technology and Carbon Emissions

Excessive fossil energy consumption is the main reason for the continuous increase in carbon dioxide emissions [24]. In addition to evaluating the direct impact of information technology progress on energy consumption, scholars have also focused directly on the effects of information technology on carbon emissions. Some scholars argue that advancements in information technology can reduce carbon emissions. Ozcan et al. (2018) used panel data to analyze the effect of information technology on CO_2_ emissions in 20 emerging economies. The long-term parametric results showed that the more internet users a country has, the lower its emissions [25]. Lu (2018) investigated the impact of information and communication technology (ICT) on carbon dioxide emissions using panel data from 12 Asian countries from 1993 to 2013. The results show that ICT negatively impacts carbon dioxide emissions [26]. Other scholars argue that the advancements in information technology will increase carbon emissions. Belkhir et al. (2018) evaluated the global carbon footprint of the entire ICT industry. They pointed out that, if not controlled, the relative contribution of ICT GHGE (Greenhouse Gas Emissions) may increase from 1–1.6% in 2007 to 14% in 2040 [27]. Park et al. (2018) used panel data from the European Union from 2001 to 2014 to examine the impact of the internet, financial development, economic growth, and trade openness on carbon dioxide emissions in selected EU (European Union) countries. The results show that electricity consumption due to Internet use positively and significantly impacts carbon emissions [28].

### 2.3. Artificial Intelligence and Energy Consumption

With the rapid development of AI, scholars have also begun to pay attention to the impact of AI on carbon emissions. In contrast to the carbon reduction effect of ICT, scholars largely agree that intelligent manufacturing brought by AI applications suppresses carbon emissions. Liu et al. (2022) investigated the impact of AI on carbon intensity using China’s industrial sector data from 2005 to 2016. The empirical results show that AI significantly reduces carbon intensity, as measured by the number of robots adopted by industry and the number of academic AI-related papers [29]. Similar to the findings of Liu et al. (2022), Li et al. (2022) empirically examined the carbon reduction effect of industrial robot applications based on the environmental Kuznets curve (EKC) model. They used data from a sample of 35 countries from 1993–2017, and the results also showed that the application of industrial robots significantly reduced carbon intensity [30]. Chen et al. (2022) assessed AI’s impact on carbon emissions based on panel data from 270 Chinese cities from 2011 to 2017, using the Bartik’s method to quantify the data of Chinese manufacturing firms and robots. The findings show that AI has a significant inhibitory effect on carbon emission intensity and that the impact of AI on carbon emission reduction varies across cities of different sizes [31].

The literature review shows that the existing studies mainly focus on three aspects. Firstly, more research focuses on the impact of information technology progress on fossil energy consumption. Most scholars believe technological progress can reduce energy consumption and improve energy use efficiency. A few scholars believe technological advancement has brought about economic expansion, increasing total energy consumption. Secondly, some studies focus directly on the impact of IT (Information Technology) progress on carbon emissions, but it is inconclusive whether IT progress has reduced carbon emissions. Thirdly, some studies have focused on the carbon reduction effect of AI, suggesting that intelligent manufacturing resulting from the applications of AI has curbed carbon emissions.

In summary, the existing research still has the following limitations. The current studies do not sufficiently explore the mechanism of carbon emission reduction achieved by the progress of information technology, and the studies on the impact of information technology progress on fossil energy consumption do not directly link fossil energy consumption with carbon emissions. However, these two problems are the core elements of the studies on the impact of intelligent manufacturing on carbon emission reduction in the industrial sectors. Given this, based on the industrial data and robot data from 2000 to 2017, this paper examines the impact of intelligent manufacturing on industrial carbon dioxide emissions and discusses its internal mechanism. The possible contributions are: first, analyzing the effect of intelligent manufacturing on emission reduction with industrial data and robot data; second, based on the overall assessment, this paper distinguishes high-carbon emission industries from low-carbon emission industries and high-intelligence industries from low-intelligence industries to examine the heterogeneous emission reduction effect of intelligent manufacturing. Third, to make up for the shortcomings of existing studies on the intrinsic mechanism of the impact of intelligent manufacturing on carbon emission reduction, this paper examines the effect of intelligent manufacturing on total fossil energy consumption and energy use efficiency. It also examines the intrinsic mechanism of intelligent manufacturing to achieve carbon emission reduction. The attempts and efforts in the above three aspects of this paper provide helpful supplements to existing studies.

## 3. Theoretical Analysis and Research Hypotheses

**Figure 2:**
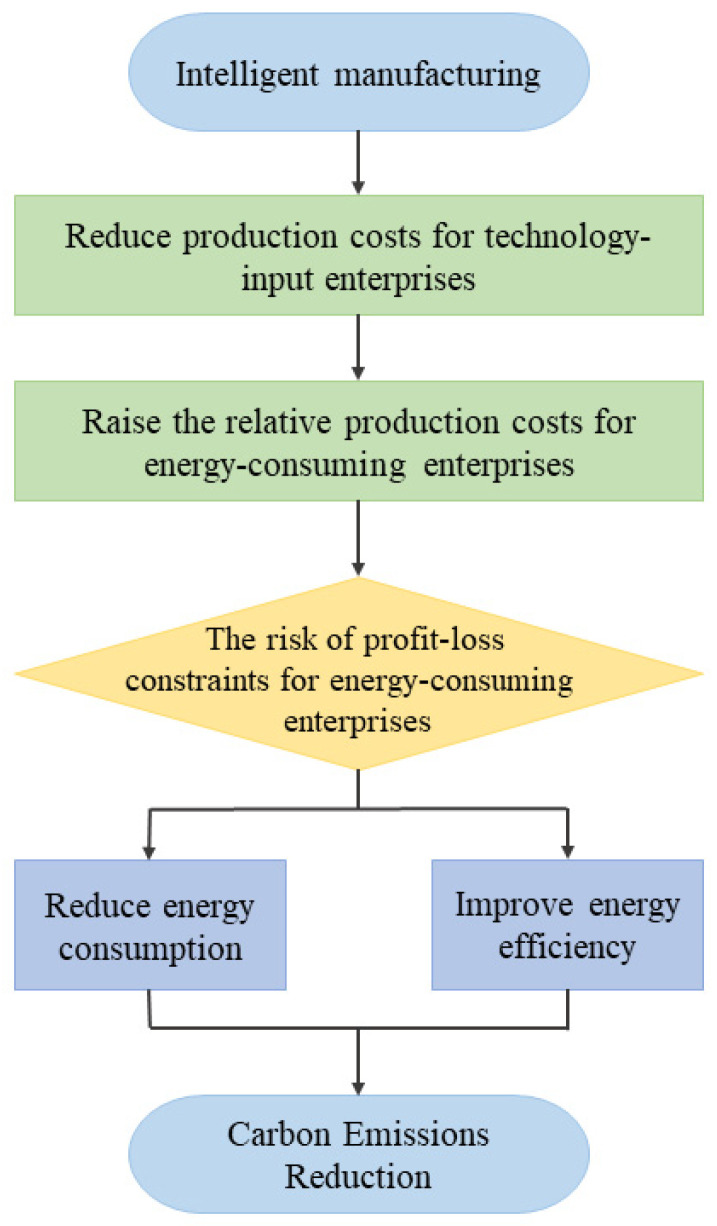
The internal mechanism of intelligent manufacturing to achieve carbon emission reduction.

The application of information technology in production has promoted the rapid development of intelligent manufacturing in various industries [32]. Can intelligent manufacturing brought by technological progress reduce carbon emissions in multiple industries to achieve carbon dioxide emission reduction? Figure 2 shows the specific internal mechanism. Firstly, technological progress causes a rebound effect, affecting the industry’s carbon dioxide emission reduction [33]. On the one hand, technological advancement has positive externalities. Technological progress can improve energy efficiency and save energy consumption, which is conducive to pushing the industry to reduce carbon dioxide emissions. On the other hand, technological progress has negative externalities. Technological progress will promote rapid economic growth, resulting in new demand for energy consumption and resistance to energy-saving energy consumption, which is not conducive to reducing carbon dioxide emissions in the industrial sectors. Secondly, the differences between industries will impact intelligent manufacturing’s carbon dioxide emission reduction [34]. Different industries have different carbon emissions, and the impact of intelligent manufacturing on high-carbon and low-carbon emission industries may be heterogeneous, and various industries have different degrees of intelligence intensity, and the effect of intelligent manufacturing on industries with higher intelligence intensity and industries with lower intelligence intensity may also be heterogeneous [35]. Combined with the above two levels of analysis, the rebound effect of technological progress and industry differences will affect the carbon emission reduction effect of intelligent manufacturing. So, it is uncertain whether intelligent manufacturing can promote carbon dioxide emission reduction in all industries. Therefore, we propose the first hypothesis:

**Hypothesis** **1.** *Intelligent manufacturing can significantly reduce CO_2_ emissions in the industrial sectors*.

Carbon dioxide emissions are closely related to total energy consumption, the proportion of fossil energy, and energy efficiency [36]. As the largest carbon dioxide emitter in the world, China is still in the process of industrialization, and energy-intensive industries still have a great demand for energy consumption [37]. China’s total energy consumption is still high, and the fossil energy consumption is the primary source. Therefore, reducing fossil energy consumption and improving the efficiency of fossil energy use is an important way to achieve CO_2_ reduction in the industrial sectors. Studies show China’s overall energy use efficiency lags behind developed countries [38]. The information technology revolution can promote the flow of production factors from energy-intensive and emission-intensive industries to highly processed and technology-intensive industries, thereby reducing fossil energy consumption and improving energy efficiency [39]. Enterprises can improve intelligent manufacturing through technological progress, thus reducing the cost of economic activities and improving production efficiency. The reduction of cost and improvement of production efficiency will also encourage enterprises to conduct technological research and development, thus enhancing the level of intelligent manufacturing, further reducing the demand for fossil energy consumption, and improving the efficiency of fossil energy use. It will have a dual effect on the industry’s fossil energy consumption, thus driving down carbon emissions. Moreover, the different levels of intelligent manufacturing in various industries will make them achieve different carbon emission reduction effects. Therefore, we have the second hypothesis:

**Hypothesis** **2.** *Intelligent manufacturing achieves carbon reduction in the industrial sectors by reducing fossil energy consumption and improving the energy use efficiency*.

## 4. Methods and Data

### 4.1. Model Setting 

This paper takes China’s industrial sector as the research object and examines the impact of intelligent manufacturing on carbon dioxide emissions in different industries based on industrial robot data. To this end, this paper establishes the following panel measurement model at the industry level:

(1) $$CO_{2}emission_{it}=\alpha +\beta \times Intelligent_{it}+\sum Control_{it}+\mu_{i}+\delta_{t}+\xi_{it}$$

In the equation, $CO_{2}emission_{it}$ represents carbon dioxide emissions. This paper uses the total industrial carbon dioxide emissions and the per capita industrial carbon dioxide emissions to measure the carbon emissions at the industry level. $Intelligent_{it}$ denotes the level of industrial robot use in industry *i* in year *t*, it says how many robots will be used by 10,000 people in this industry during this period. It will measure the degree of intelligent manufacturing in different industries; $Control_{it}$ represents a series of control variables that affect the industry carbon emissions. $\mu_{i}$ and $\delta_{t}$ represent industry fixed effect and time fixed effect, $\xi_{it}$ is the random disturbance term. *β* is the top concern of this paper, it is used to evaluate the net effect of industrial robot usage level on industrial carbon dioxide emissions, and this paper expects high robot usage level will have a positive emission reduction effect.

### 4.2. Variables Selection

Carbon dioxide emission is the focus of this study. To comprehensively examine the carbon dioxide emissions of the industrial sectors, this paper first chooses the total industrial carbon emissions as the main object and compares it with the per capita industrial carbon emissions. Then, considering the fact that fossil energy consumption in all energy consumption types will generate more carbon emissions in industrial sectors, it is essential to investigate the carbon emissions caused by different energy consumption in the industrial sectors, which is highly policy-oriented. Given this, to analyze the impact of intelligent manufacturing on carbon emissions of different types of energy consumption, this paper also selects the carbon emissions of coal consumption, oil consumption, and natural gas consumption as the explained variables to supplement the comprehensive analysis.

In addition, as for the core explanatory variable, this paper chooses the robot usage per 10,000 people in the industrial sector as the measurement index of intelligent manufacturing. Aside from the above main variables, according to the industry’s carbon emissions characteristics, this paper also selects factors such as industrial scale, export value, industry gross output value, foreign investment, and industry profit to control the industry’s output scale. It shows detailed together with descriptive statistical analysis in Table 1.

### 4.3. Data Sources

**Figure 3:**
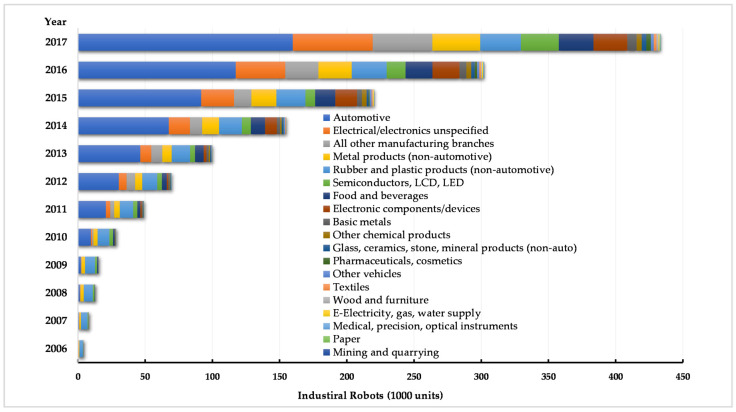
The number of robots used in different industries in China. Source: International Federation of Robotics (IFR).

In order to examine the impact of intelligent manufacturing on industry carbon emissions, this paper constructs Chinese industry-level panel data from international industrial robot statistics and relevant data collected from the China Energy Statistical Yearbook and the China Statistical Yearbook over the years to conduct the study. The specific data sources are as follows: First, this paper uses robotics-related statistics to capture the scale of robot applications in China from the industry level to measure intelligent manufacturing. International industrial robot statistics come from IFR, a database that provides authoritative data on industrial robot applications worldwide broken down by application area, industry branch, and robot type (http://ifr.org/worldrobotics/ (accessed on 1 November 2022)). Based on these data, this paper constructs the sizeable number of new robots per year by industry. Limited by the availability of robotics data, the sample interval of this paper is 2012–2017. Secondly, the paper’s core is to examine the impact of intelligent manufacturing on carbon emission reduction in the industrial sectors. The measurement of CO_2_ mainly depends on energy consumption, so the total consumption of natural gas, total consumption of crude oil, and total consumption of coal in 42 industries from 2000–2017 was searched and compiled in the China Energy Statistical Yearbook to calculate the industry-level CO_2_ emissions. Finally, considering other important factors affecting the development of the industry, a series of industry-level related economic variables were therefore selected, which were obtained from the China Statistical Yearbook for all years. Figure 3 shows the number of robots used in different industries in China.

## 5. Empirical Results and Analysis

### 5.1. The Impact of Intelligent Manufacturing on Carbon Emissions

To examine the impact of intelligent manufacturing on carbon emissions, we first estimate model (1) regression to assess the effects of industry robot use on carbon emissions. The specific estimation results are shown in Table 2. Among them, columns (1) and (3) are the regression results without adding control variables, while columns (2) and (4) are the estimated results after adding the relevant control variables affecting carbon emissions. It shows that intelligent manufacturing can significantly reduce carbon dioxide emissions in the manufacturing industry, both in terms of total and per capita carbon emissions. Its reduction effect is significant at the 1% and 5% confidence levels. Meanwhile, comparing the assessed coefficients shows that intelligent manufacturing can significantly reduce carbon emissions per capita by about 23% and total carbon emissions by 11%, which shows that intelligent manufacturing has a more substantial effect on carbon emissions per capita, and hypothesis 1 is verified.

The regression results in Table 2 show that intelligent manufacturing significantly reduces the industry CO_2_ emissions, but how does intelligent manufacturing affect the carbon emissions from fossil energy sources in industry production? To further complement and refine this conclusion, we analyze the impact of intelligent manufacturing on fossil energy carbon emissions in industry production by breaking down the carbon dioxide emissions caused by different fossil energy sources. The regression results are shown in Table 3. Columns (1) and (2) in Table 3 mainly analyze the effect of intelligent manufacturing on carbon emissions caused by coal consumption. Columns (3) and (4) focus on the impact of intelligent manufacturing on carbon emissions caused by oil-based consumption, while columns (5) and (6) focus on carbon emissions caused by natural gas consumption. It shows that intelligent manufacturing reduces carbon dioxide emissions from coal and oil consumption in the industry at a 5% significance level and reduces carbon dioxide emissions from natural gas use in the industry at a 1% significance level. Moreover, the assessed coefficients further indicate that intelligent manufacturing has the most significant dampening effect on carbon emissions released from coal consumption, reducing them by about 9.44%. In contrast, it has a weaker impact on carbon emissions from oil and natural gas consumption. This result may be due to the characteristics of China’s manufacturing industry in terms of energy use, which is overly dependent on coal consumption and less on natural gas. The results in Table 3 strongly suggest that intelligent manufacturing can reduce carbon emissions from the industrial sectors’ three primary fossil energy sources, but with significant variability in reducing carbon emissions from different energy sources.

### 5.2. Heterogeneity Analysis of the Impact of Intelligent Manufacturing on Carbon Emissions

The previous regression results show that intelligent manufacturing can reduce the industrial sectors’ total and per capita carbon emissions and can positively affect the reduction of carbon emissions generated by fossil energy in the industry. However, due to the existence of industry differences, the carbon emission levels of different industries and the degree of intelligence intensity of various industries vary. This section will analyze the heterogeneity of intelligent manufacturing affecting industry carbon dioxide emissions.

#### 5.2.1. The Impact of Intelligent Manufacturing on Carbon Emissions under Industrial Carbon Emission Differences

According to the difference in industrial carbon emissions, this paper divides the industries that produce carbon emissions into high-carbon and low-carbon emission industries to analyze the heterogeneous impact of intelligent manufacturing on carbon emissions. The regression results are shown in Table 4. It shows that in industries with high-carbon emissions, intelligent manufacturing significantly reduces the total carbon emissions and per capita carbon emissions at the significance level of 1%, reducing the total carbon emissions and per capita carbon dioxide emissions by about 12.37% and 11.79%. However, in low-carbon emission industries, intelligent manufacturing does not significantly affect the carbon emission level of the industry. It may be because high-carbon emission industries urgently need more space for carbon emission reduction than low-carbon emission industries, and the emergence of intelligent manufacturing can have a significant effect on high-carbon emission industries but will not have a substantial impact on low-carbon industries.

#### 5.2.2. The Impact of Intelligent Manufacturing on Carbon Emissions under Industrial Intelligence Differences

Since different industries have different degrees of intelligence, this paper divides the industries that generate carbon emissions into those with high-intelligence intensity and those with low-intelligence intensity according to the differences in the application of robots in the industries so as to study the impact of the use of robots on carbon emissions under the intelligence differences. The regression results are shown in Table 5. The regression results show that intelligent manufacturing significantly reduces carbon dioxide emissions at the 5% significance level in the more intelligent industries and significantly reduces carbon dioxide emissions per capita at the 1% significance level. In contrast, in the less intelligent industries, intelligent manufacturing does not significantly impact the industry’s carbon emission level. It may be because the more intelligent industries are more likely to achieve intelligent manufacturing than the less intelligent industries. The more intelligent industries can take advantage of the better intelligent infrastructure to change production methods and improve production efficiency, thus achieving significant carbon emission reduction in the industry. In contrast, the less intelligent industries may not require much robot input and not change the original industry production methods on energy consumption or energy efficiency, and thus cannot achieve significant emission reduction effects in the industry.

### 5.3. Robustness Test

The empirical results demonstrate that intelligent manufacturing can significantly reduce the industry’s CO_2_ emissions. To ensure the robustness of the results, we will conduct relevant robustness tests on the regression results in this section. Specifically, the robustness tests include indicator replacement, reducing the sample period, eliminating interference policy, instrumental variable method, and adding control variables.

#### 5.3.1. Indicator Replacement

The above analysis focuses on the industry’s total and per capita carbon dioxide emissions, which are measured more from the perspective of the total amount. However, in reality, we are not only concerned about the total carbon emissions but also the carbon emissions per unit of output value. Therefore, to avoid the bias of carbon emission measurement in this paper, the carbon emission of the industry is re–measured here, and Table 6 shows measured regression results. Among them, column (1) measures carbon emissions per unit of output value by the ratio of total carbon dioxide emissions to the industry’s total output value. The regression results show that intelligent manufacturing still positively affects reducing carbon emissions per unit of output value, and the impact is significant at the 1% confidence level. Column (2) measures the carbon emissions of individual enterprises by the ratio of total carbon dioxide emissions to the number of enterprises in the industry. The regression results show that Intelligent manufacturing also has a significant positive impact on carbon emissions per unit enterprise. The regression results are shown in columns (3)–(5) of Table 6. It shows that regardless of whether added control variables, intelligent manufacturing still has a significant positive impact on reducing overall carbon emissions and carbon emissions per unit of output value. The regression results in Table 6 are consistent with those in Table 2, indicating that the above results are more robust.

#### 5.3.2. Reducing the Sample Period

The main study period of this paper focuses on 2000–2017 but, considering that this paper is mainly based on the use of robots to measure intelligent manufacturing, and these data are less available between 2000 and 2004, we are concerned that this lack of data may have an impact on the results of this paper. Given this, the study period of the sample is adjusted here by year reduction. Table 7 shows the reduced regression results. Among them, columns (1) and (4) selected the sample from 2005–2017, columns (2) and (5) are from 2009–2017, and columns (3) and (6) are from 2010–2017. The regression results all show that intelligent manufacturing has a significant inhibitory effect on reducing total and per capita carbon emissions. This result is consistent with the regression results in Table 2, further validating that this finding that intelligent manufacturing has a significant inhibitory effect on CO_2_ emissions is robust.

#### 5.3.3. Eliminating Interference Policy

Since the Chinese government implemented a series of environmental policies after 2000, implementing these will undoubtedly have a particular impact on the industry’s carbon emissions, which will indirectly affect the assessment of this paper. Given this, we exclude three policies the Chinese government has implemented. First, at the beginning of the 21st century, due to the excessive energy consumption and environmental pressure in China, the government introduced a series of policies to curb the development of the “two high and one leftover” industries. In particular, in 2006, the “two high and one leftover” industries moved towards sustainable development through industrial transformation and cleaner production under the macro–control of national policies. To exclude the influence of national policies on the robustness of the regression results, we exclude the “two high and one leftover” industries from the industry sample that are easily influenced by national policies for robustness testing. Second, the Chinese government’s five-year plan will include the support policies of the relevant industries within five years. To avoid the regression results being affected by the support policies of the five–year plan, the authors remove the industries affected by the support policies of the five-year plan from the industry sample for the robustness test, and the regression results are shown in columns (2) and (5) of Table 8. Finally, the Chinese government has conducted carbon pilots in the thermal power, gas, paper, and tobacco industries to mitigate climate change. To avoid the impact of the carbon pilot policy, the authors exclude the thermal power, gas, paper, and tobacco industries from the industry sample for robustness testing, and the regression results are shown in columns (3) and (6) in Table 8. The specific regression results show that after excluding the above significant environmental policies, the regression results all indicate that intelligent manufacturing significantly positively affects total and per capita carbon emissions.

#### 5.3.4. Instrumental Variable Method

The potential endogeneity of the econometric model is also a problem that needs to be considered in this paper. Since the core explanatory variable in this paper is the number of robots in the industry, there is a correlation between the number of robots used and carbon emissions. At the same time, industries with higher carbon emissions may have a more vigorous production capacity and thus preferentially promote the use of industrial robots. This interaction may lead to a probable mutual causality between the model’s independent and explanatory variables. Therefore, in this paper, we choose instrumental variables to be addressed here. The choice of instrumental variables generally requires that the instrumental variables are highly correlated with the independent variables and cannot be correlated with the random disturbance terms. Under this constraint, we choose the average level of robot use worldwide as the instrumental variable for robot use in Chinese industries. It is obvious that there is a correlation between these two variables.

In contrast, the average number of robots used in industries around the world bears no correlation between the number of robots used and China’s carbon emissions, so the instrumental variable is satisfied, and the specific regression results are shown in Table 9. It shows that the average number of robots in all countries as an instrumental variable passes the under–identification and weak identification tests. It also shows the first stage results that the instrumental variable is positively correlated with the core explanatory variable intelligent machines at the 1% significance level, which indicates the rationality of the instrumental variable selected in this paper. From the regression results of the second stage, IV-2SLS is consistent with the conclusion obtained from OLS that intelligent manufacturing has a significant inhibitory effect on carbon emissions, and the absolute value of the coefficient is more significant, which further indicates the reliability of the primary conclusion of this paper.

#### 5.3.5. Adding Control Variables

In addition to the above robustness tests, we try to control more industry-level economic variables in the model to bring more factors affecting CO_2_ emissions under control to ensure the net effect of intelligent manufacturing involving carbon emissions. Here we select essential variables such as industry gross output value, the number of employees, industry tax burden paid total corporate liabilities, and industry Chinese–owned capital into the econometric model and run regressions, as shown in Table 10. Among them, subsets (1)–(5) in Table 10 are added to the model, while (6) is a regression with all macro factors included in the econometric model. The results show that even after controlling for the above five essential elements, intelligent manufacturing still positively affects reducing carbon emissions. The effect is significant at the 5% confidence level, proving the reliability of the previous regression results.

## 6. Mechanism Test of Intelligent Manufacturing to Achieve Emission Reduction Effect

The above analysis provides detailed evidence and rich robustness tests on China’s intelligent manufacturing’s carbon emission reduction effect. The findings fully demonstrate that the large-scale use of robots can reduce carbon dioxide emissions in the industry. Then, the question that makes us ponder is why intelligent manufacturing can achieve carbon emission reduction and its mechanism. 

Changes in enterprise behavior can profoundly impact its carbon emissions. Therefore, we speculate that the mechanism underlying the significant reduction of carbon emissions by intelligent manufacturing focuses more on the impact of intelligent manufacturing on enterprise behavior. There are differences in the application of intelligent manufacturing among enterprises in different industries. Intelligent manufacturing can promote a rapid increase in output scale and a significant increase in revenue for IT-based enterprises. However, it raises the relative cost of energy-consumption-based traditional industries, leading to the risk of revenue loss for energy-consumption-based traditional enterprises under competitive market conditions. Under the loss risk constraint due to intelligent manufacturing, enterprises will adjust their energy use and efficiency.

We first examine the impact of intelligent manufacturing on enterprise income. The data on enterprise income are difficult to obtain, so in order to measure the impact of intelligent manufacturing on the income of enterprises in the industry, we choose two indicators, the number of enterprises in the industry with losses and the proportion of the number of enterprises with losses to the total number of enterprises, respectively, and the specific results are shown in Table 11. Among them, columns (1) and (2) mainly examine the impact of intelligent manufacturing on the number of enterprises in the industry. The results show that intelligent manufacturing increases the number of enterprises in the market. Due to the increase in the number of enterprises, the competition within the industry increases, which leads to enterprises facing higher competitiveness. Columns (3)–(6) examine the effect of intelligent manufacturing on the number of loss–making enterprises and the share of the number of loss–making enterprises in the total number of enterprises. The results show that intelligent manufacturing exacerbates enterprises’ losses within traditional industries dominated by energy consumption. Thus, intelligent manufacturing forces firms to face the dual pressure of increased external competition and revenue losses.

Figure 2 shows that enterprises will change their energy use and efficiency. Therefore, we speculate that intelligent manufacturing can affect carbon emissions by reducing fossil energy consumption and improving energy use efficiency. Given this, we first analyze the relationship between intelligent manufacturing and energy consumption, and the specific results are shown in Table 12. Among them, column (1) in Table 12 mainly analyzes the impact of intelligent manufacturing on total energy consumption, and the results show that the improvement of intelligence level can reduce the energy consumption of the industry by about 7.48%, and this reduction effect is significant at the 5% confidence level. In addition to the analysis of total energy consumption, we also analyze different types of fossil energy consumption, including coal consumption, coke consumption, oil consumption, and natural gas consumption, as shown in columns (2)–(5) in Table 12. From the results of the regression, we can see that the increase in the level of intelligent energy reduces the consumption of different types of fossil energy, but the impact of intelligent manufacturing on the total consumption of different types of fossil energy varies, with the most significant effect of intelligent manufacturing on reducing the total consumption of coal, the second most significant impact on the consumption of coke and natural gas, and the most negligible significant impact on the total consumption of oil.

In addition to energy consumption, improving energy efficiency is also an effective way to reduce carbon emissions. Therefore, to examine the impact of intelligent manufacturing on energy use efficiency, this paper uses intelligent manufacturing to regress the overall energy use efficiency and the three major fossil energy use efficiencies separately. The specific results are shown in Table 13. Among them, column (1) is the ratio of total energy consumption to the total industrial output value, column (2) is the ratio of total energy consumption to industrial value–added, and columns (3)–(5) are the ratios of total coal, oil, and natural gas consumption to the total industrial output value, respectively. The results show that intelligent manufacturing is conducive to improving energy use efficiency and the efficiency of the three major fossil energy sources, and intelligent manufacturing has the most significant impact on improving the efficiency of coal use, followed by oil, and the most negligible impact on improving the efficiency of natural gas use. Through the above analysis, hypothesis 2 is tested, and intelligent manufacturing can reduce carbon emissions in the industry by reducing fossil energy consumption and improving the efficiency of fossil energy use.

In summary, intelligent manufacturing can inhibit fossil energy consumption and improve energy efficiency. It has the most apparent inhibitory effect on coal consumption and the most noticeable improvement in coal use efficiency. This variability in the impact of intelligent manufacturing on fossil energy may be related to China’s long–established coal-dominated energy consumption structure. Coal, oil, and gas are China’s primary source of fossil energy consumption and brings more than 90% of carbon emissions from the three primary fossil energy consumptions. The impact of intelligent manufacturing on coal–led fossil energy consumption is conducive to promoting the improvement and upgrading of China’s energy consumption structure, which further reduces fossil energy carbon emissions.

## 7. Conclusions

With the rapid development of information technology, the application of intelligent manufacturing has been promoted in various production fields. Intelligent manufacturing is essential in reducing carbon emissions and can effectively promote China’s goal of achieving a carbon peak by 2030 and carbon neutrality by 2060. Therefore, it is crucial to explore the impact of intelligent manufacturing on carbon emissions and its internal mechanism. Based on this, this paper uses industry data and robot data from 2000 to 2017 to test the impact of intelligent manufacturing on carbon emissions and analyze the effects of intelligent manufacturing on carbon emissions in heterogeneous industries. The study’s main conclusions are as follows: intelligent manufacturing positively impacts the industry’s total carbon emissions and carbon emissions per capita. Intelligent manufacturing can comprehensively reduce the carbon emissions from the consumption of the three primary fossil energies in the industrial sectors. Heterogeneity analysis shows that intelligent manufacturing can significantly reduce emissions in high-carbon emission industries than in low–carbon emission industries. Furthermore, intelligent manufacturing can reduce carbon emissions in industries with high-intelligence intensity but not in industries with low– intelligence intensity. 

The mechanism analysis shows that intelligent manufacturing leads to the risk of loss for energy-based companies. Under the income loss constraint, enterprises will adjust the amount of energy used and energy efficiency. Intelligent manufacturing can significantly reduce the total consumption of various types of fossil energy. However, the degree of the reduction varies for different kinds of fossil energy. In general, intelligent manufacturing has the most apparent impact on reducing the total consumption of coal, followed by coke and natural gas consumption, and the most negligible effect on total oil consumption. Intelligent manufacturing is also conducive to improving the energy efficiency of the three primary fossil energies. It has the most obvious impact on improving coal use efficiency, followed by oil and natural gas. So, we can see that intelligent manufacturing can reduce carbon emissions by reducing fossil energy consumption and improving energy efficiency. The policy implications of the above conclusions are as follows.

(1) The government should use technological advances to drive production changes to ensure innovation-driven green development. Intelligent manufacturing can reduce the industry’s carbon emissions, so the potential for technological innovation needs to be further explored to provide a lasting driver for the green development of production. The government should further increase policy support and investment in innovative research and development to support enterprises in developing advanced energy-saving and environmental protection technologies, processes, and equipment. At the same time, relevant management departments should actively carry out pilot demonstrations in process intelligence, aggregating the multi-dimensional strength of manufacturing enterprises and research institutes to achieve interdisciplinary and cross-disciplinary collaborative research and support traditional industries to achieve carbon emission reduction with intelligent production.
(2) The government should focus on developing high-carbon emission and less intelligent–intensive industries and promote low carbonization with intelligent manufacturing. Intelligent manufacturing can comprehensively reduce carbon emissions from fossil energy consumption in the industry. However, due to the heterogeneity of its impact on carbon emission reduction in the industrial sectors and the current status of high energy consumption in China’s high carbon emission industries, less-intelligent intensive industries urgently need to upgrade industry technology and renewal industry equipment. Therefore, the government should control the development of high energy–consuming, heavy chemical industries and other high carbon emission enterprises and adjust their products and industrial structures. Less intelligent and less intensive enterprises should take the initiative to seek technological upgrading and transformation of production methods.
(3) The government should improve China’s energy consumption structure with intelligent manufacturing in the industrial sectors and gradually establish an energy system centered on renewable energy. As a sizeable coal-consuming country, China has formed an energy consumption structure dominated by coal consumption. Adopting targeted emission reduction policies is necessary to change this high–carbon emission energy consumption structure. Intelligent manufacturing has a significant effect on both reducing fossil energy consumption and improving fossil energy use efficiency. Therefore, it is necessary to use intelligent manufacturing to improve the energy consumption structure by improving energy efficiency and establishing a renewable energy–centered energy consumption system in light of the current situation of China’s energy consumption.

## Figures and Tables

**Table 1:** Descriptive statistics of main variables.

Variable	Calculation Method	Obs	Mean	Std. Dev.
CO_2_ emissions	ln (total carbon emissions of the industry + 1)	718	2.7256	1.8076
CO_2_ emissions per capita	ln ((total carbon emissions of the industry + 1)/(number of employees in the industry + 1))	621	−2.0527	1.5854
Coal CO_2_ emissions	ln (industry coal consumption carbon emissions + 1)	718	2.1633	1.6759
Oil CO_2_ emissions	ln (industry oil consumption carbon emissions + 1)	696	0.1805	0.4075
Natural gas CO_2_ emissions	ln (industry natural gas consumption carbon emissions + 1)	756	0.6082	0.9996
Intelligent	ln (number of robots in industry + 1)	756	0.1851	0.9379
size	ln (total industry assets + 1)	756	7.7773	3.0553
profit	ln (total industry profit + 1)	752	5.2761	2.4744
open	ln (industry export delivery value + 1)	756	4.6751	3.1278
fdi	ln (industry foreign capital + 1)	756	3.8204	2.5969
energy	ln (total energy consumption by industry + 1)	756	6.9990	2.4705
state	ln (industry state capital + 1)	756	4.3180	2.5034
labor	ln (number of employees in the industry + 1)	756	4.0827	2.0721
tax	ln (tax payable by industry + 1)	756	3.6631	2.7391
debt	ln (total industry liabilities + 1)	756	7.2298	2.9051
gdp	ln (gross industrial output value + 1)	756	5.8686	4.1352

**Table 2:** The impact of intelligent manufacturing on carbon emissions under different types of emission indicators.

	CO_2_ Emissions		CO_2_ Emissions per Capita	
	(1)	(2)	(3)	(4)
Intelligent	−0.1160 **	−0.1100 **	−0.2552 ***	−0.2299 ***
	(0.0433)	(0.0459)	(0.0548)	(0.0590)
Control variables	NO	YES	NO	YES
Time fixed effect	YES	YES	YES	YES
Industry fixed effect	YES	YES	YES	YES
_cons	2.7384 ***	2.0874 ***	−2.0225 ***	1.6683 ***
	(0.0048)	(0.2983)	(0.0061)	(0.5827)
N	718	715	620	617
F	7.184	3.308	21.718	30.265
r2_a	0.958	0.959	0.921	0.936

Note: (1) The values in parentheses are robust standard errors of clustering; (2) **, and *** indicate significance at the confidence levels of 5% and 1%, respectively.

**Table 3:** The Impact of Intelligent Manufacturing on Different Types of Emission Sources.

	Coal CO_2_ Emissions		Oil CO_2_ Emissions		Natural Gas CO_2_ Emissions	
	(1)	(2)	(3)	(4)	(5)	(6)
Intelligent	−0.0981 **	−0.0944 **	−0.0636 **	−0.0655 **	−0.0321 ***	−0.0332 ***
	(0.0447)	(0.0444)	(0.0297)	(0.0306)	(0.0101)	(0.0108)
Control variables	NO	YES	NO	YES	NO	YES
Time fixed effect	YES	YES	YES	YES	YES	YES
Industry fixed effect	YES	YES	YES	YES	YES	YES
_cons	2.1741 ***	1.6738 ***	0.1874 ***	0.1284 *	0.6142 ***	0.4581 **
	(0.0049)	(0.2513)	(0.0032)	(0.0639)	(0.0019)	(0.1966)
N	718	715	696	693	756	752
F	4.816	2.248	4.575	1.879	10.064	1.893
r2_a	0.962	0.964	0.825	0.819	0.881	0.877

Note: (1) The values in parentheses are robust standard errors of clustering; (2) *, **, and *** indicate significance at the confidence levels of 10%, 5% and 1%, respectively.

**Table 4:** The impact of intelligent manufacturing on carbon emissions under carbon emission differences.

	High-Carbon Emission Industries		Low-Carbon Emission Industries	
	CO_2_ Emissions	CO_2_ Emissions per Capita	CO_2_ Emissions	CO_2_ Emissions per Capita
	(1)	(2)	(3)	(4)
Intelligent	−0.1237 ***	−0.1179 ***	2.2606	37.1189
	(0.0254)	(0.0281)	(58.8531)	(54.1533)
Control variables	YES	YES	YES	YES
Time fixed effect	YES	YES	YES	YES
Industry fixed effect	YES	YES	YES	YES
_cons	2.3461 ***	3.5478	2.1571 ***	1.7443 ***
	(0.2940)	(2.1168)	(0.2936)	(0.4770)
N	267	231	448	386
F	6.573	11.848	5.132	24.742
r2_a	0.995	0.990	0.950	0.915

Note: (1) The values in parentheses are robust standard errors of clustering; (2) *** indicate significance at the confidence levels of 1%.

**Table 5:** The impact of intelligent manufacturing on carbon emissions under the intelligence differences.

	Industries with High-Intelligence Intensity		Industries with Low-Intelligence Intensity	
	CO_2_ Emissions	CO_2_ Emissions per Capita	CO_2_ Emissions	CO_2_ Emissions per Capita
	(1)	(2)	(3)	(4)
Intelligent	−0.3319 **	−0.6605 ***	0.0114	−0.0975
	(0.1341)	(0.1423)	(0.0476)	(0.0597)
Control variables	YES	YES	YES	YES
Time fixed effect	YES	YES	YES	YES
Industry fixed effect	YES	YES	YES	YES
_cons	3.6975 ***	−0.5822	0.1168	1.5940 ***
	(0.1950)	(1.3370)	(0.2471)	(0.5287)
N	410	348	302	265
F	2.866	11.940	11.663	50.723
r2_a	0.966	0.959	0.743	0.920

Note: (1) The values in parentheses are robust standard errors of clustering; (2) **, and *** indicate significance at the confidence levels of 5% and 1%, respectively.

**Table 6:** Indicator replacement.

	CO_2_ Emissions per Unit Output Value	Average CO_2_ Emissions per Enterprise	CO_2_ Emissions	CO_2_ Emissions	Carbon Emissions per Unit Output Value
	(1)	(2)	(3)	(4)	(5)
Intelligent	−0.1285 ***	−0.1841 ***	−0.1016 ***	−0.1170 ***	−0.4720 ***
	(0.0457)	(0.0521)	(0.0165)	(0.0216)	(0.1016)
Control variables	YES	YES	NO	YES	YES
Time fixed effect	YES	YES	YES	YES	YES
Industry fixed effect	YES	YES	YES	YES	YES
_cons	0.0329	−1.6101 ***	6.8857 ***	6.2580 ***	5.5216 **
	(0.4883)	(0.4068)	(0.0038)	(0.5339)	(2.0892)
N	654	654	411	408	408
F	123.502	27.014	37.738	13.634	50.541
r2_a	0.964	0.950	0.961	0.962	0.954

Note: (1) The values in parentheses are robust standard errors of clustering; (2) **, and *** indicate significance at the confidence levels of 5% and 1%, respectively.

**Table 7:** Reducing the sample period.

	CO_2_ Emissions			CO_2_ Emissions per Capita		
	(1)	(2)	(3)	(4)	(5)	(6)
Intelligent	0.1240 ***	−0.0985 ***	−0.0872 **	−0.1741 ***	−0.1232 ***	−0.1045 ***
	(0.0385)	(0.0340)	(0.0334)	(0.0427)	(0.0325)	(0.0302)
Control variables	YES	YES	YES	YES	YES	YES
Time fixed effect	YES	YES	YES	YES	YES	YES
Industry fixed effect	YES	YES	YES	YES	YES	YES
_cons	1.9012 ***	2.1936 ***	2.3328 ***	1.4975 **	3.0504 ***	3.7572 ***
	(0.4128)	(0.2903)	(0.2261)	(0.6689)	(0.6079)	(0.5783)
N	475	357	317	403	294	257
F	5.661	3.954	3.920	24.185	33.568	47.325
r2_a	0.985	0.989	0.990	0.978	0.984	0.987

Note: (1) The values in parentheses are robust standard errors of clustering; (2) **, and *** indicate significance at the confidence levels of 5% and 1%, respectively.

**Table 8:** Eliminating interference policy.

	CO_2_ Emissions			CO_2_ Emissions per Capita		
	(1)	(2)	(3)	(4)	(5)	(6)
Intelligent	−0.1566 ***	−0.1124 **	−0.1179 **	−0.2793 ***	−0.2248 ***	−0.2116 ***
	(0.0461)	(0.0443)	(0.0440)	(0.0868)	(0.0677)	(0.0512)
Control variables	YES	YES	YES	YES	YES	YES
Time fixed effect	YES	YES	YES	YES	YES	YES
Industry fixed effect	YES	YES	YES	YES	YES	YES
_cons	1.8434 ***	2.0838 ***	2.6753 ***	1.4433 **	1.4615 **	1.1187
	(0.3057)	(0.3170)	(0.3011)	(0.5639)	(0.6743)	(0.8469)
N	447	537	520	364	450	449
F	3.180	3.792	3.422	25.059	22.809	13.420
r2_a	0.943	0.958	0.959	0.920	0.933	0.939

Note: (1) The values in parentheses are robust standard errors of clustering; (2) **, and *** indicate significance at the confidence levels of 5% and 1%, respectively.

**Table 9:** Instrumental variable test.

	CO_2_ Emissions		CO_2_ Emissions per Capita	
	(1)	(2)	(3)	(4)
Intelligent	−0.6193 **	−0.6611 **	−1.3020 ***	−1.1136 ***
	(0.2791)	(0.2816)	(0.4038)	(0.3639)
Control variables	NO	YES	NO	YES
Time fixed effect	YES	YES	YES	YES
Industry fixed effect	YES	YES	YES	YES
N	706	703	620	617
F	7.939	7.241	2.957	6.977
First stage				
Iv	0.420 ***	0.423 ***	0.416 ***	0.411 ***
	(0.091)	(0.093)	(0.099)	(0.100)
LM statistic Chi-sq (1)	5.85	6.81	16.2	13.82
Anderson-Rubin Wald test	21.13	20.86	17.65	17.04
Cragg-Donald Wald F statistic	21.24	20.79	17.67	16.89

Note: (1) The values in parentheses are robust standard errors of clustering; (2) **, and *** indicate significance at the confidence levels of 5% and 1%, respectively.

**Table 10:** Adding control variables.

	CO_2_ Emissions	CO_2_ Emissions	CO_2_ Emissions	CO_2_ Emissions	CO_2_ Emissions	CO_2_ Emissions
	(1)	(2)	(3)	(4)	(5)	(6)
Intelligent	−0.1093 **	−0.1116 **	−0.1071 **	−0.1043 **	−0.1070 **	−0.0954 **
	(0.0451)	(0.0463)	(0.0449)	(0.0478)	(0.0473)	(0.0471)
gdp	0.0037					0.0018
	(0.0197)					(0.0183)
labor		0.0136				0.0247
		(0.0275)				(0.0296)
tax			0.0349			0.0480 **
			(0.0263)			(0.0235)
debt				0.3510		0.4486
				(0.4467)		(0.4543)
state					−0.0261	−0.0507 *
					(0.0272)	(0.0281)
Control variables	YES	YES	YES	YES	YES	YES
Time fixed effect	YES	YES	YES	YES	YES	YES
Industry fixed effect	YES	YES	YES	YES	YES	YES
_cons	2.0841 ***	2.0920 ***	2.0775 ***	2.1584 ***	2.0997 ***	2.1956 ***
	(0.3050)	(0.2978)	(0.2911)	(0.3174)	(0.3154)	(0.3339)
N	715	715	715	715	715	715
F	2.931	2.955	2.889	3.706	3.328	3.040
r2_a	0.959	0.959	0.959	0.960	0.959	0.960

Note: (1) The values in parentheses are robust standard errors of clustering; (2) *, **, and *** indicate significance at the confidence levels of 10%, 5% and 1%, respectively; (3) The test results of CO_2_ emissions per capita do not show significant changes, due to space limitations, the corresponding results are not presented here.

**Table 11:** The impact of intelligent manufacturing on enterprise income.

	Total Number of Enterprises	Total Number of Enterprises	Total Number of Loss-Making Enterprises	Total Number of Loss-Making Enterprises	Total Number of Loss-Making Enterprises/Total Number of Enterprises	Total Number of Loss-Making Enterprises/Total Number of Enterprises
	(1)	(2)	(3)	(4)	(5)	(6)
Intelligent	0.2521 ***	0.0310 *	0.3689 **	0.2916 **	0.0134 ***	0.0103 ***
	(0.0464)	(0.0165)	(0.1530)	(0.1366)	(0.0025)	(0.0020)
Control variables	NO	YES	NO	YES	NO	YES
Time fixed effect	YES	YES	YES	YES	YES	YES
Industry fixed effect	YES	YES	YES	YES	YES	YES
_cons	7.4175 ***	−0.2699	3.7599 ***	1.0213	0.1024 ***	−0.0033
	(0.0086)	(0.4066)	(0.0283)	(1.1401)	(0.0005)	(0.0277)
N	756	752	756	752	756	752
F	29.501	224.425	5.812	12.184	28.567	16.562
r2_a	0.871	0.990	0.846	0.877	0.758	0.837

Note: (1) The values in parentheses are robust standard errors of clustering; (2) *, **, and *** indicate significance at the confidence levels of 10%, 5% and 1%, respectively.

**Table 12:** The impact of intelligent manufacturing on energy consumption.

	Total Energy Consumption	Total Coal Consumption	Total Coke Consumption	Total Oil Consumption	Total Natural Gas Consumption
	(1)	(2)	(3)	(4)	(5)
Intelligent	−0.0748 **	−0.1427 ***	−0.1326 ***	−0.0768 **	−0.1019 **
	(0.0285)	(0.0307)	(0.0332)	(0.0327)	(0.0378)
Control variables	YES	YES	YES	YES	YES
Time fixed effect	YES	YES	YES	YES	YES
Industry fixed effect	YES	YES	YES	YES	YES
_cons	5.7509 ***	4.9926 ***	5.1362 ***	1.8222 ***	−1.5469
	(0.6859)	(0.7194)	(0.5889)	(0.4911)	(1.1048)
N	612	612	636	752	590
F	2.075	6.547	5.282	3.424	3.935
r2_a	0.956	0.938	0.919	0.925	0.903

Note: (1) The values in parentheses are robust standard errors of clustering; (2) **, and *** indicate significance at the confidence levels of 5% and 1%, respectively.

**Table 13:** The impact of intelligent manufacturing on energy efficiency.

	Total Energy Efficiency	Total Energy Efficiency	Coal Use Efficiency	Oil Use Efficiency	Natural Gas Use Efficiency
	(1)	(2)	(3)	(4)	(5)
Intelligent	−0.2282 **	−0.2161 *	−0.3708 **	−0.3140 ***	−0.2827 **
	(0.1120)	(0.1071)	(0.1367)	(0.1061)	(0.1374)
Control variables	YES	YES	YES	YES	YES
Time fixed effect	YES	YES	YES	YES	YES
Industry fixed effect	YES	YES	YES	YES	YES
_cons	−1.0195	−0.8782	4.2199 ***	0.7517	−0.3974
	(0.9410)	(1.4954)	(1.5219)	(1.7090)	(1.7172)
N	752	752	639	425	593
F	66.556	37.142	15.256	31.676	18.914
r2_a	0.922	0.908	0.941	0.956	0.950

Note: (1) The values in parentheses are robust standard errors of clustering; (2) *, **, and *** indicate significance at the confidence levels of 10%, 5% and 1%, respectively.

## Data Availability

Publicly available datasets were analyzed in this study. These data can be found here: National Bureau of Statistics. Available online: https://data.stats.gov.cn/ (accessed on 1 November 2022); International Federation of Robotics. Available online: http://ifr.org/worldrobotics/ (accessed on 1 November 2022).
